# Correction: A specific sequence in the genome of respiratory syncytial virus regulates the generation of copy-back defective viral genomes

**DOI:** 10.1371/journal.ppat.1008099

**Published:** 2019-10-03

**Authors:** Yan Sun, Eun Ji Kim, Sébastien A. Felt, Louis J. Taylor, Divyansh Agarwal, Gregory R. Grant, Carolina B. López

There is an error in the accession number for the RSV isolate used in Fig 6, “A conserved rejoin region determines cbDVG formation in infected human patients”, as well as in its caption. Please see the correct Fig 6 and the complete, correct Fig 6 caption here.

**Fig 6:**
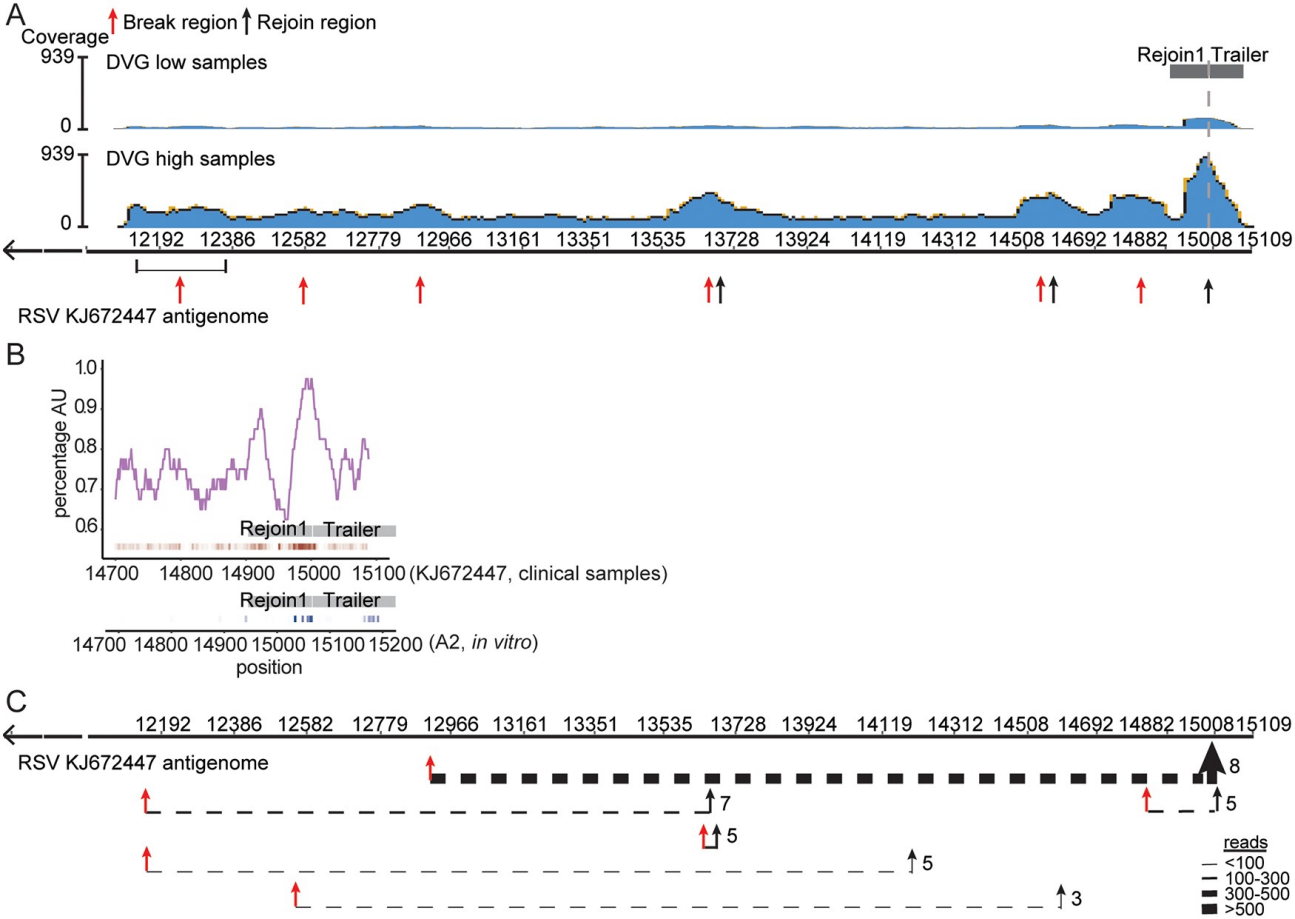
A conserved rejoin region determines cbDVG formation in infected human patients. (A) Four specimens from RSV A-positive pediatric patients with low content of DVGs and 6 with high content of DVGs were selected for RNA-seq. All seq reads that passed the quality threshold were analyzed by VODKA to screen for cbDVG junctions. DVG junction reads were then aligned to the RSV reference genome NCBI KJ672447. Blue histogram shows a synopsis of total coverage at any given position of the RSV reference genome (illustrated by the y-axis). The number on the right side of the graph represents the total reads at the position with highest coverage. (B) AU-content (top, purple) within nucleotides 14700–15109 of the RSV strain A isolate KJ672447 (human samples) is plotted centered on a 40 base-pair sliding window in both plots. Grey box indicated the position of Rejoin1 and Trailer. Individual rejoin points identified by DVG junction reads from pediatric samples within this region are represented by red ticks underneath the graph. The rejoin points from *in vitro* infection of RSV stocks 1–6 are plotted using blue ticks. The darker color represented increased rejoin frequency at a given position. (C) Top 6 major DVGs identified from clinical samples are depicted by dashed lines that join their break points (red arrows) and rejoin points (black arrows). The thickness of the line represents the frequency of DVG reads at a given DVG as indicated. The number of patients that have the particular DVG is indicated at the right of the rejoin point for each cbDVG.
