# Supplementary figures and images for: A specific sequence in the genome of respiratory syncytial virus regulates the generation of copy-back defective viral genomes
Source: PLoS Pathog. 2019 Apr 17;15(4):e1007707. doi: 10.1371/journal.ppat.1007707 (PMC6504078; doi:10.1371/journal.ppat.1007707)

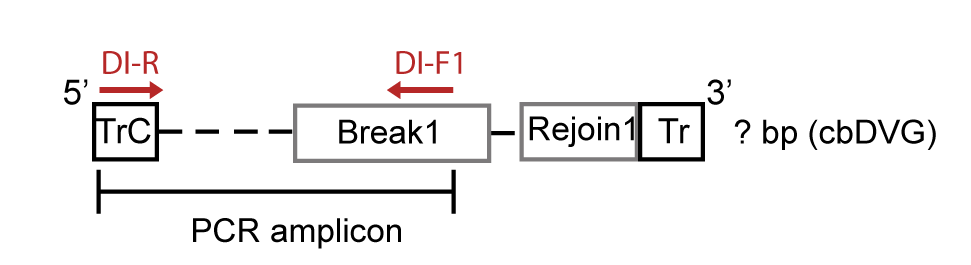

Supplement: S1 Fig — Diagram of a representative cbDVG generated from the minigenome Pair1 construct with unknown length. Red arrows indicate the location of primers used for PCR to detect DVGs. This strategy allows detection of cbDVGs of various sizes as indicated by the dashed line. (TIF) [file ppat.1007707.s001.tif]

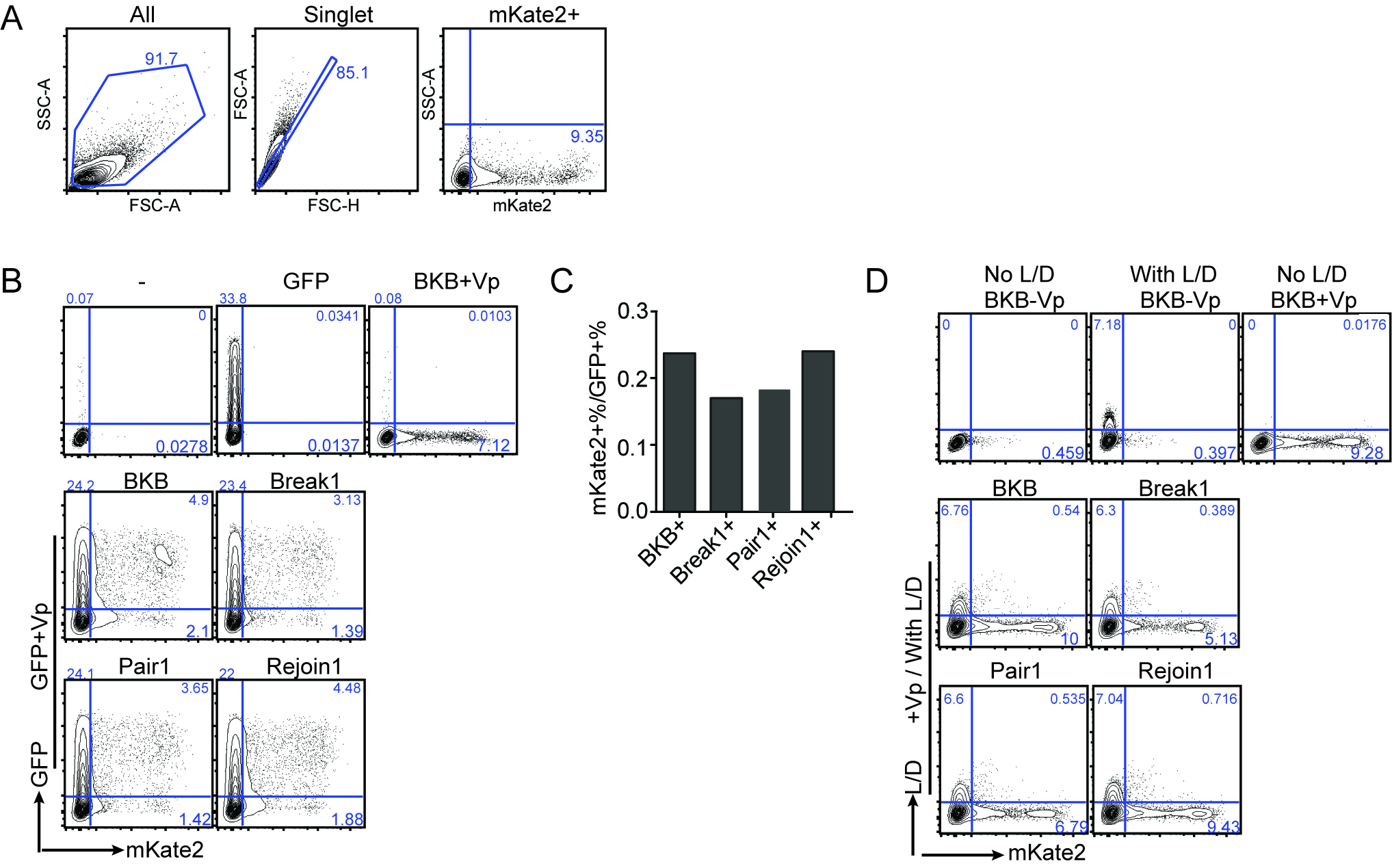

Supplement: S2 Fig — (A) Gating strategy for all flow cytometry data shown in Figs 2 and 4A and S2. (B-C) BSR-T7 cells were co-transfected with 4 helper plasmids expressing the polymerase proteins, pmax-GFP, and the designated minigenome construct. mKate2 expression and GFP expression were measured by flow cytometry. Representative flow plots are shown in (B) and fold change in (C). (D) BSR-T7 cells were co-transfected with the four helper plasmids and the designated minigenome construct. Cells were first stained with Live/Dead aqua followed by flow cytometry. Representative flow plots are shown. (TIF) [file ppat.1007707.s002.tif]

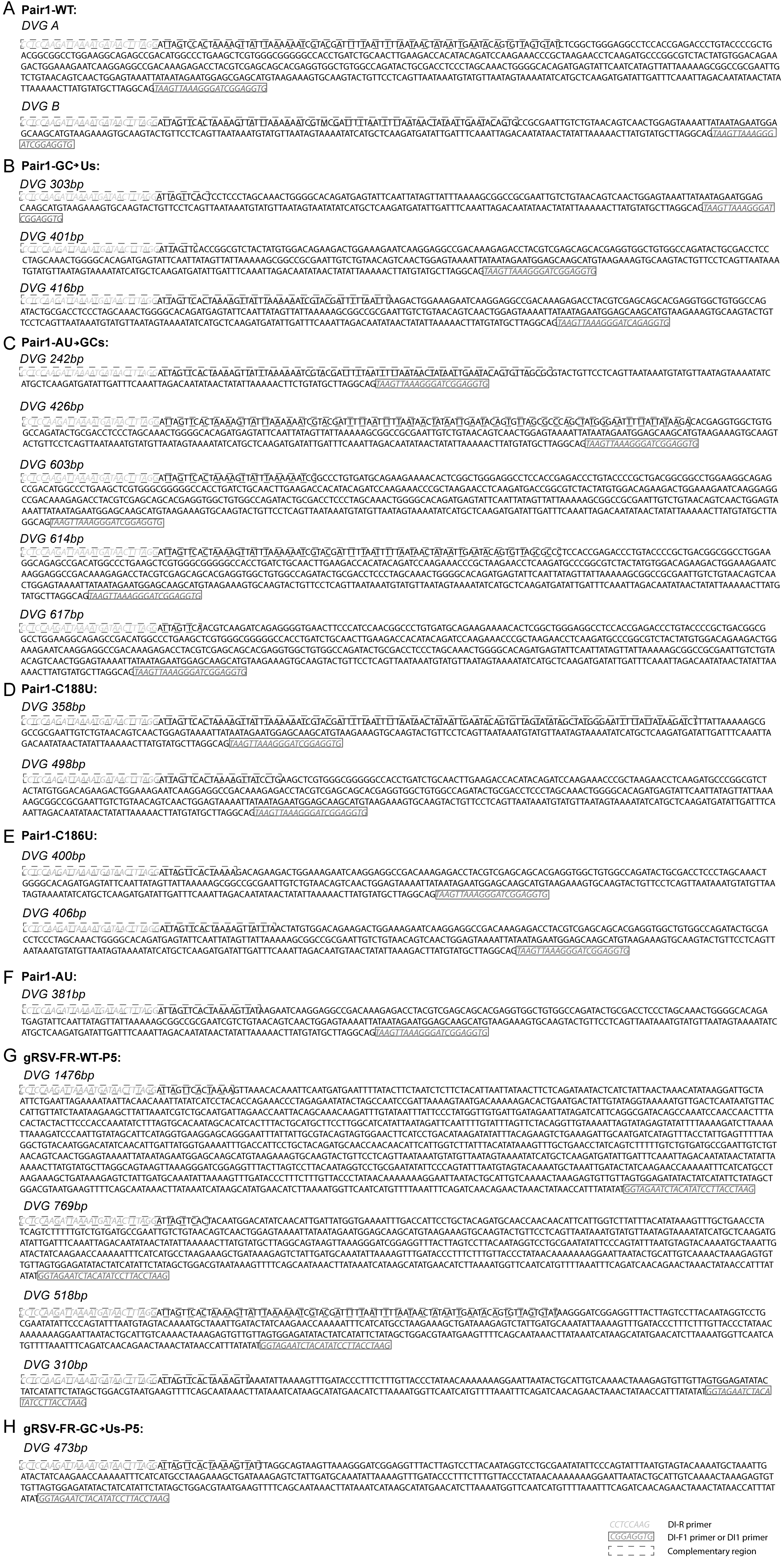

Supplement: S3 Fig — Sequences of cbDVGs identified from labeled bands in RSV minigenome system (A-F), and in gRSV-FR-WT (G) or gRSV-FR-GC >Us viral infections (H). (TIF) [file ppat.1007707.s003.tif]

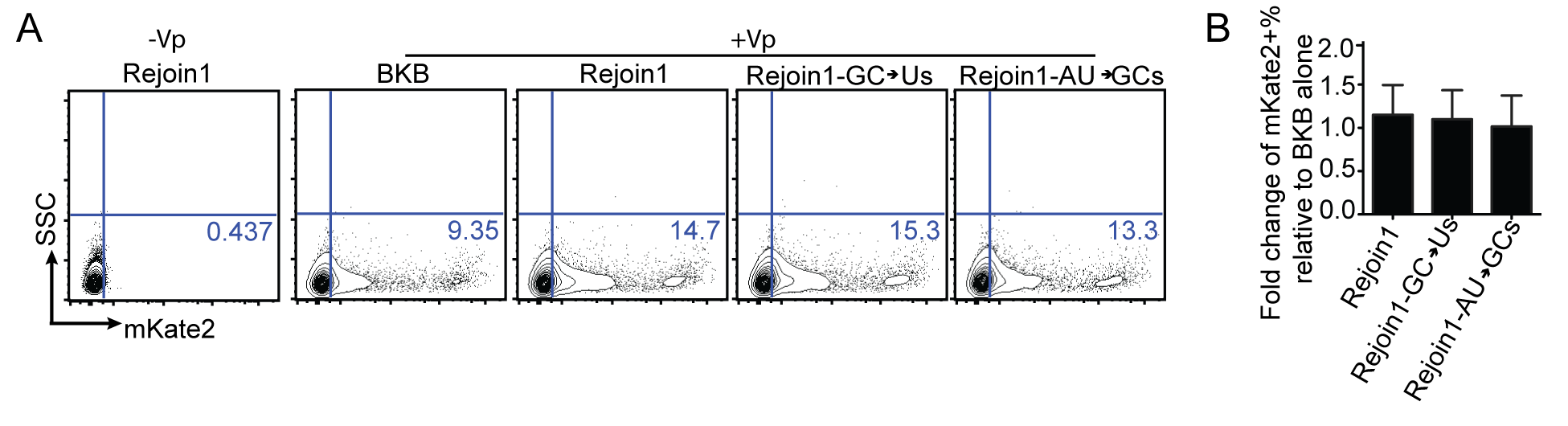

Supplement: S4 Fig — The same mutations contained in the Pair1 construct were introduced in the Rejoin1 construct. BSR-T7 cells were co-transfected with all 4 helper plasmids as well as BKB or Rejoin1 mutations. mKate2 expression was measured by flow cytometry. Representative flow plots are shown in panel (A), and quantification of three repeats is shown as fold change in (B). Fold change was calculated as the percentage of mKate2 expressing cells transfected with Rejoin1 or its two mutants over BKB control. (TIF) [file ppat.1007707.s004.tif]

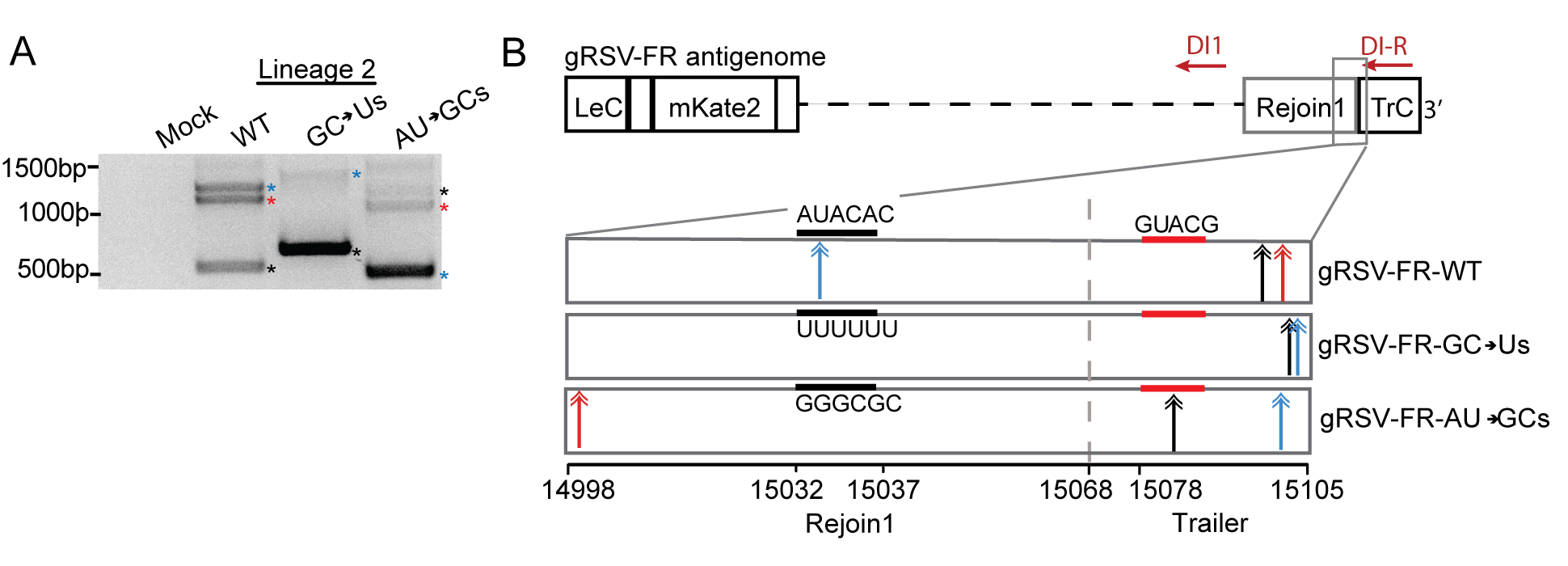

Supplement: S5 Fig — gRSV-FR-WT and gRSV-FR-GC->Us viruses were independently propagated from P0 to P3 in HEp2 cells to generate lineage 2 (L2). gRSV-FR-AU>GCs was generated and passaged to P5 the same way as WT and Us viruses. (A) cbDVG detection from viral stocks gRSV-FR-AU>GCs-P5, gRSV-FR-WT-P3 (L2), and gRSV-FR-GC>Us-P3 (L2) using RT-PCR with DI1/DI-R primer set in HEp2 cells. Confirmed cbDVG-like amplicons by Sanger sequencing are labeled by asterisks. Different colors correspond to the color of double arrows in (B). (B) Schematic summary of all identified cbDVG rejoin points in (A). (TIF) [file ppat.1007707.s005.tif]

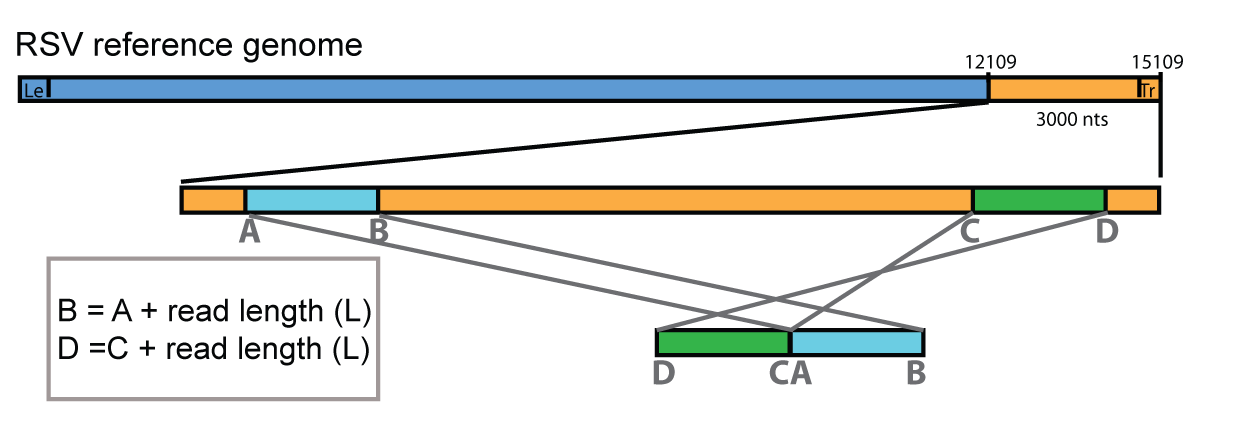

Supplement: S6 Fig — Schematic representation of how VODKA constructs a theoretic library for cbDVGs from the last 3000 nucleotides (nts) of the genome. (TIF) [file ppat.1007707.s006.tif]
