# Supplementary material for: A specific sequence in the genome of respiratory syncytial virus regulates the generation of copy-back defective viral genomes
Source: PLoS Pathog. 2019 Apr 17;15(4):e1007707. doi: 10.1371/journal.ppat.1007707 (PMC6504078; doi:10.1371/journal.ppat.1007707)
Supplement: S1 Table — (DOCX) [file ppat.1007707.s007.docx]

**S1 Table. Oligo primer list.**

| **Primer name** | **Sequences (5’-3’)** | **Application in paper** |
| --- | --- | --- |
| DI1 (RSV) | CTTAGGTAAGGATATGTAGATTCTACC | DVG-RT-PCR |
| DI-R | CCTCCAAGATTAAAATGATAACTTTAGG | DVG-RT-PCR |
| SeV DI1 | GGTGAGGAATCTATACGTTATAC | DVG-RT-PCR |
| gSeV DI1 | ACCAGACAAGAGTTTAAGAGATATGTATT | DVG-RT-PCR |
| DI-F1 | CACCTCCGATCCCTTTAACTTA | DVG-RT-PCR |
| DI-F2 | CAATATCATCTTGAGCATGATATTTTAC | DVG-RT-PCR |
| DI-F3 | CATTATTCATTATGAAAGTTGTATAACAGACTAC | DVG-RT-PCR |
| RSV G-F | AACATACCTGACCCAGAATC | qPCR |
| RSV G-R | GGTCTTGACTGTTGTAGATTGCA | qPCR |
| *GAPDH-F* | CTCCCACTCTTCCACCTTCG | qPCR |
| *GAPDH-R* | CCACCACCCTGTTGCTGTAG | qPCR |
| T7 term-R | CCGG*GAATTC*AACATATAGTTCCTCCTTTCAGCA | Cloning |
| RSV TrC37-F | GCGC*ACTAGT*GACCT*GGGTCCC*TTAG*GCTCTTC*TTAAAAATCGTACGATTTTTTAAATAACTTTTAGTGAAC | Cloning |
| T7 prom-F | GCGC*ACTAGT*TAATACGACTCACTATAGGGAAGTTT | Cloning |
| mKateGE-R | CCG*GGGACCC*CTAA*GCGGCCGC*TTTTTAATAACTATAATTGAATACTCATCTGTGCC | Cloning |
| TrC+Rejoin1-F | GCGCA*GGGTCCC*TTAGG*CCATGG*AGCTAG*GCTCTTC*AAAACTGATTAAAATCACAGGTAGTCTGTTATAC | Cloning |
| Break1-F | GGCCA*GCGGCCGC*GAATTGTCTGTAACAGTCAACTGG | Cloning |
| Break1-R | CGCGA*GGGTCCC*ACCTCCGATCCCTTTAACTTACT | Cloning |
| Rejoin1-F | GGCCA*GGGTCCC*AAAACTGATTAAAATCACAGGTAGTCTG | Cloning |
| Rejoin1-R | CGCGA*GCTCTTC*TTTAATTTTTAATAACTATAATTGAATACAGTGTTAGTG | Cloning |
| GCs->Us-F | AAAAATCGTACGATTTTTAATTTTTAATAACTATAATTGAATACAGTGTTAAAAAAAAGCTATGGGAATTTTTATTATAAGATCTTTATTCATTATTCATTATGAAAG | Site-mutagenesis |
| GCs->Us-R | CTTTCATAATGAATAATGAATAAAGATCTTATAATAAAAATTCCCATAGCTTTTTTTTAACACTGTATTCAATTATAGTTATTAAAAATTAAAAATCGTACGATTTTT | Site-mutagenesis |
| AU->GCs-F | GTACGATTTTTAATTTTTAATAACTATAATTGAATACAGTGTTAGCGCCCAGCTATGGGAATTTTTATTATAAGATCTTTATTCATTATTCATTA | Site-mutagenesis |
| AU->GCs-R | TAATGAATAATGAATAAAGATCTTATAATAAAAATTCCCATAGCTGGGCGCTAACACTGTATTCAATTATAGTTATTAAAAATTAAAAATCGTAC | Site-mutagenesis |
| C1284T-F | GAATAAAGATCTTATAATAAAAATTCCCATAGCTATATACTAACACTGTATTCAATTATAGTTATTAAAAA | Site-mutagenesis |
| C1284T-R | TTTTTAATAACTATAATTGAATACAGTGTTAGTATATAGCTATGGGAATTTTTATTATAAGATCTTTATTC | Site-mutagenesis |
| C1286T-F | TTAATTTTTAATAACTATAATTGAATACAGTGTTAATGTATAGCTATGGGAATTTTTATTATAAGATCTTT | Site-mutagenesis |
| C1286T-R | AAAGATCTTATAATAAAAATTCCCATAGCTATACATTAACACTGTATTCAATTATAGTTATTAAAAATTAA | Site-mutagenesis |
| mut virus PmlI fwd | TTAACACGTGGTGAGAGAGGACCCACTAAA | Site-mutagenesis for virus |
| mut virus MulI rev | GCTTACGCGTATATAGTTCCTCCTTTCAGCAAAA | Site-mutagenesis for virus |
| A mut virus rev | CTATAATTGAATACAGTGTTAAAAAAAAGCTATGGGAATTTTTATTATAAGATC | Site-mutagenesis for virus |
| A mut virus fwd | GATCTTATAATAAAAATTCCCATAGCTTTTTTTTAACACTGTATTCAATTATAG | Site-mutagenesis for virus |
